# 943 variants identified synthesizing chromatin conformation capture and quantitative trait loci with results from the Alzheimer’s Disease Variant Portal study

**DOI:** 10.1002/alz.089276

**Published:** 2025-01-03

**Authors:** Jeffrey Cifello, Pavel P. Kuksa, Li‐San Wang, Yuk Yee Leung

**Affiliations:** ^1^ Penn Neurodegeneration Genomics Center, Perelman School of Medicine, University of Pennsylvania, Philadelphia, PA USA; ^2^ Penn Neurodegeneration Genomics Center, Dept of Pathology and Laboratory Medicine, University of Pennsylvania, Philadelphia, PA USA; ^3^ University of Pennsylvania Perelman School of Medicine, Philadelphia, PA USA

## Abstract

**Background:**

Recent genetic studies have implicated >70 genomic loci associated with the risk for Alzheimer’s Disease. However, the underlying functional mechanisms remain unclear. Several functional genomics (FG) methods such as chromosome conformation (CC) capture technologies and expression quantitative trait loci (eQTLs) have been developed to study the genetic targets. In this study, we aimed at using a large collection of brain eQTL and CC datasets to analyze/identify targets for all of the genetic signals in Alzheimer’s Disease Variant Portal (ADVP), a curated and harmonized database of the main genetic findings from > 200 AD GWAS studies.

**Method:**

Using the Harmonization and Integration Pipeline for Functional Genomics (hipFG), >12,000 eQTL and >400 chromatin interaction datasets (>43M interactions) were harmonized, indexed to allow for rapid genomic search. After filtering for non‐cell‐line derived brain tissues and cell types, 78 eQTL and 9 chromatin interaction datasets remained (25 cell types/tissues). Chromatin interactions were analyzed further if one anchor overlapped with a QTL variant, and the other anchor overlapped with the QTL’s target gene’s promoter. These introduced QTL‐target and interaction pairs. Next, we performed linkage‐disequilibrium (LD) pruning and expansion on all genome‐wide significant variants from studies of non‐Hispanic Whites (NHW) in ADVP and evaluated overlaps with chromosome interactions and eQTLs.

**Result:**

Across 1125 curated ADVP variants (p<5e‐8), 90.84% are from NHW, representing 301 independent signals. We LD‐expanded these to 11,964 unique variants. We observed that 10,888 of these variants (91%) overlap with eQTLs, 5156 (43.1%) overlap with chromatin interaction anchors, and 943 (7.9%) overlap with both QTL‐target and CC pairs. Of these 943 variants, only 14.2% are eQTLs for a nearest gene. The 188 eQTL target genes and 271 genes overlapping interaction anchors combined for 359 unique genes, and pathway analyses on this combined set was significantly enriched for amyloid‐beta metabolism and transmembrane transport. These results are in line with previous findings in the field.

**Conclusion:**

Our analyses showed that using diverse functional annotations enables the discovery of new target genes not found in one assay alone. The ADVP variant regulated genes (supported by both eQTLs and chromatin interactions) implicated known disease mechanisms.

